# Prospective study of vitrectomy for epiretinal membranes in patients with good best-corrected visual acuity

**DOI:** 10.1186/s12886-019-1185-z

**Published:** 2019-08-14

**Authors:** Hiroyuki Nakashizuka, Yorihisa Kitagawa, Yu Wakatsuki, Koji Tanaka, Koichi Furuya, Takayuki Hattori, Ryusaburo Mori, Hiroyuki Shimada

**Affiliations:** Division of Ophthalmology, Department of Visual Sciences, Nihon University School of Medicine, Nihon University Hospital, 1-6 Kandasurugadai, Chiyoda-ku Tokyo, 101-8309 Japan

**Keywords:** Epiretinal membrane, Good visual acuity, Metamorphopsia, Aniseikonia, Vitrectomy

## Abstract

**Background:**

To evaluate the efficacy of epiretinal membrane removal in patients with good best-corrected visual acuity (BCVA) for improving visual function and quality of life (QOL).

**Methods:**

This prospective case study compared 37 subjects with preoperative BCVA ≦ 0.046 logMAR (Good group) to 35 patients with 0.10–0.52 logMAR (Moderate group) at 3 and 6 months. Linear mixed-effect models were used for statistical analysis. The primary outcome was the horizontal metamorphopsia score (MH) at 6 months postoperatively (post-6 M), while secondary outcomes were postoperative BCVA, vertical metamorphopsia score (MV), aniseikonia, stereopsis and central foveal thickness. In the Good group, QOL was assessed using the National Eye Institute Visual Functioning Questionnaire-25 (NEI VFQ-25) at 6 and 12 months.

**Results:**

MH was significantly improved at post-3 M and post-6 M in the both groups but there were no significant differences between the two groups. MV showed no improvement at the final observation in either group. LogMAR BCVA was significantly improved at post-6 M in the Good group, which had significantly better vision than the Moderate group. Preoperative vertical and horizontal aniseikonia scores remained unchanged in the Good group at post-6 M but worsened in the Moderate group. The NEI VFQ-25 score improved in the Good group, reflecting improved general health, general vision, and mental health.

**Conclusions:**

Early epiretinal surgery for patients with BCVA ≦ 0.046 logMAR was effective for improvement of HM, BCVA, and QOL and prevented worsening of aniseikonia.

**Trial registration:**

UMIN000021220. Registered 10 September 2015.

UMIN Clinical Trials Registry.

## Background

Epiretinal membrane is a translucent tissue that develops on the retinal surface and is reportedly present in 7 - 11.8% individuals age 40 years and older [1, 2]. Epidemiological studies conducted on the Japanese population have found that 4.0 - 5.4% of individuals have epiretinal membranes, indicating that aging is a risk factor [3, 4]. A recent study utilizing optical coherence tomography showed an epiretinal membrane to be present in 8.6% of post-cataract surgery patients with a mean age of 74.9 years [5]. Thus, patients with epiretinal membrane will increase as the population ages.

Although the epiretinal membrane itself does not cause blindness, symptoms of metamorphopsia and aniseikonia will develop. Furthermore, best-corrected visual acuity (BCVA) will decrease with the progression of these clinical conditions. It has been reported that preoperative BCVA is associated with postoperative BCVA prognosis [6, 7], though patients with lower preoperative BCVA can obtain higher improvement rates [8]. Therefore, epiretinal membrane is not generally treated surgically in patients with good BCVA. Recently, important visual functions other than BCVA, including metamorphopsia, aniseikonia, and binocular vision, have received increasing attention. Okamoto et al reported that metamorphopsia has a greater effect on vision-related quality of life (QOL) than BCVA [9].

Micro-incision vitrectomy surgery has recently been introduced and was shown to increase the safety of retinal surgery [10]. Therefore, cases with good BCVA, i.e. ≦ 0.046 logMAR (decimal BCVA, 0.9), are also candidates for surgery. Even in recent studies [6, 9, 11–15], the averages of preoperative logMAR BCVA have ranged from 0.17 to 0.7, while reports of good BCVA (≦ 0.046 logMAR) are very rare [16].

The present study aimed to elucidate the efficacy of epiretinal membrane removal in patients with good BCVA for improving visual function and QOL.

## Methods

The present study was conducted in accordance with the tenets of the Declaration of Helsinki following approval from the Institutional Review Board of Nihon University Hospital (Tokyo, Japan). Written informed content was obtained from all patients before enrollment.

This prospective case series included a total of 37 patients with epiretinal membrane with good BCVA (≦0.046 logMAR; decimal BCVA, 0.9–1.5; Good group) enrolled between December 2015 and September 2016. Data from these patients were compared with retrospective data obtained from 35 cases (moderate group) whose BCVA was measured at 0.10–0.52 logMAR (decimal BCVA, 0.3–0.8) between April 2015 and April 2016 at 3 and 6 months (M) postoperatively (post-3 M and post-6 M, respectively).

The following data were acquired: quantitative assessment of metamorphopsia using M-CHARTS® (Inami Co., Tokyo, Japan) [17], quantitative assessment of aniseikonia using the New Aniseikonia test (Handaya Co., Tokyo, Japan.) [18], stereopsis assessment using the Titmus Stereo test (TST; Stereo Optical Co., Inc.), and assessment of visual function using decimal BCVA measurements. Central foveal thickness (CFT) was measured using optical coherence tomography (OCT; Spectralis®, Heidelberg Engineering Inc., Heidelberg, Germany). Decimal BCVA data were converted to logMAR scores for statistical processing. Logarithmic transformation was performed on the TST results. The primary and secondary outcome measures are listed below. Because the Moderate group data were retrospective, only post-3 M and post-6 M were available, while in the Good group these data were measured prospectively at post-1 M, post-3 M, post-6 M, and post-12 M. Furthermore, patient satisfaction levels were assessed in the Good group only using the National Eye Institute Visual Functioning Questionnaire-25 (VFQ-25) [19].

### Primary outcome measure

Horizontal metamorphopsia score (MH) at post-6 M.

### Secondary outcome measures

i) Decimal BCVA (logMAR score) preoperatively and at post-1 M, post-3 M, and post-12 M.
ii) MH preoperatively and at post-1 M, post-3 M, and post-12 M.
iii) Vertical metamorphopsia score (MV) preoperatively and at post-1 M, post-3 M, post-6 M, and post-12 M.
iv) Horizontal aniseikonia score (AH) preoperatively and at post-1 M, post-3 M, post-6 M, and post-12 M.
v) Vertical aniseikonia score (AV) preoperatively and at post-1 M, post-3 M, post-6 M, and post-12 M.
vi) Stereopsis measured by TST preoperatively and at post-1 M, post-3 M, post-6 M, and post-12 M.
vii) CFT measured by OCT preoperatively and at post-1 M, post-3 M, post-6 M, and post-12 M.
viii) NEI VFQ-25 score preoperatively and at post-6 M, and post-12 M.

Statistical analysis included a mixed model to compare chronological changes in all assessed data for both the Good and the Moderate group. A multivariate model with intentional selection was used to analyze variables. Notably, a mixed model (trend model with observation time points used as continuous quantities) was applied, with postoperative MH as the response variable, factors, observation time points, and interactions detected at the observation time points; independent variables as the fixed effects; and patients as the random effect to assess the effects exerted by preoperative variables (e.g., age, sex, pseudo-macular hole, BCVA, CFT, MH, MV, AH, and AV) on postoperative MH changes.

The model including the baseline MH obtained through variable selection was a mixed one, with the month of observation serving as the category. This mixed model was used to estimate the mean value for MH at each month of observation with the baseline value.

SAS program v.9.4 (SAS Institute, Cary, NC) was employed. Statistical significance was set at *P* < 0.05. Exclusion criteria were as follows: i) a previous history of vitrectomy, ii) ocular inflammation, iii) retinal vascular diseases, iv) cataracts influencing BCVA and v) more than 2.0 diopters of anisometropia before and after surgery.

#### Surgical procedure

In all patients, surgery was performed by the same surgeon (H.N.) using a 27-gauge vitrectomy system (CONSTELLATION® Vision System, Alcon Japan Ltd.) under retrobulbar anesthesia. All patients ≥50 years of age with phakic eyes underwent phacoemulsification and intraocular lens (IOL) implantation. Following core vitrectomy, a micro-hooked needle was created using a 27-G needle, and the epiretinal membrane was removed. Subsequently, the inner limiting membrane was removed following staining with 0.0625% brilliant blue G. After peripheral vitrectomy, approximately 30% of the vitreous cavity was replaced with air to accelerate the self-sealing of the sclerotomies. The posterior lens capsule was opened using a vitreous cutter to prevent posterior capsule opacification. The sclerotomies were then confirmed to have no leaking, and in patients with closure failure, a single 8–0 absorbable suture (coated 8–0 vicryl, ETHICON) was placed. In patients with a pseudo-macular hole, 100% fluid-air exchange was performed, and patients were required to lie in the prone position for 12–24 h.

## Results

### Background characteristics

Patient background characteristics are presented in Table 1. There were no significant differences in sex, age, or percentage of pseudo-macular holes between the two groups. In the Good and Moderate groups, two and one eye, respectively, had already undergone IOL implantation. In the Good group, one eye had been treated with lens sparing vitrectomy.

No postoperative complications such as vitreous hemorrhage, retinal detachment, endophthalmitis, visual field loss, cystoid macular edema, and cataract progression were reported.

**Table 1:** Patient background characteristics

	Good group (*n* = 37)	Moderate group (*n* = 35)	*P*-value
Sex (M: F)	17:20	13:22	0.48
Age (years ± SD)	64.1 ± 7.4	66.7 ± 7.8	0.15
Pseudo-macular hole (number of eyes)	11	9	0.8

*M* male, *F* female

### BCVA (Table 2 and Fig. 1)

Significant improvements in BCVA at each time point were noted in both the Good and the Moderate group. The Good group, however, showed significantly better BCVA preoperatively, as well as at post-3 M and post-6 M (*P* < 0.0001, P < 0.0001, and *P* = 0.003, respectively).

**Table 2:** Parameter values for the Good and Moderate groups

	Good group									Moderate group				
	Pre-op	Post-1 M		Post-3 M		Post-6 M		Post-12 M		Pre-op	Post-3 M		Post-6 M	
		Mean ± SD	*P*-value	Mean ± SD	*P*-value	Mean ± SD	*P*-value	Mean ± SD	*P*-value	Mean ± SD	Mean ± SD	P-value	Mean ± SD	*P*-value
logMAR BCVA	−0.09 ± 0.08	− 0.12 ± 0.09	0.08	− 0.13 ± 0.10	0.02	−0.13 ± 0.10	0.01	− 0.13 ± 0.07	0.01	0.23 ± 0.11	− 0.01 ± 0.14	< 0.0001	− 0.06 ± 0.13	< 0.0001
MH	0.84 ± 0.60	0.52 ± 0.63	0.0003	0.44 ± 0.58	< 0.0001	0.45 ± 0.59	< 0.0001	0.36 ± 0.50	< 0.0001	0.87 ± 0.66	0.65 ± 0.70	0.01	0.50 ± 0.60	< 0.0001
MV	0.86 ± 0.57	0.57 ± 0.53	0.006	0.66 ± 0.66	0.05	0.62 ± 0.60	0.02	0.79 ± 0.69	0.38	0.79 ± 0.60	0.48 ± 0.47	0.004	0.59 ± 0.63	0.06
AH	3.03 ± 3.02	2.78 ± 2.89	0.68	2.61 ± 2.72	0.49	2.81 ± 2.89	0.71	3.17 ± 3.83	0.82	3.60 ± 4.55	5.11 ± 4.58	0.01	5.50 ± 3.89	0.009
AV	2.78 ± 2.62	2.56 ± 2.65	0.69	2.56 ± 3.06	0.69	2.96 ± 3.13	0.75	3.14 ± 3.34	0.52	4.09 ± 4.88	5.20 ± 4.71	0.05	5.56 ± 4.39	0.01
TST	1.92 ± 0.39	1.96 ± 0.55	0.45	1.95 ± 0.46	0.6	1.98 ± 0.57	0.32	1.93 ± 0.52	0.88	2.19 ± 0.55	2.12 ± 0.51	0.17	2.02 ± 0.44	0.01
CFT	388.4 ± 111.5	376.2 ± 78.9	0.3	349.5 ± 73.8	0.001	338.8 ± 73.7	< 0.0001	329.1 ± 63.3	< 0.0001	485.7 ± 158.8	402.7 ± 82.1	< 0.0001	381.2 ± 85.5	< 0.0001

*BCVA* best-corrected visual acuity, *MH* horizontal metamorphopsia score, *MV* vertical metamorphopsia score, *AH* horizontal aniseikonia score, *AV* vertical aniseikonia score, *TST* Titmus Stereo test, *CFT* central foveal thickness, *SD* standard deviation

**Fig. 1:**
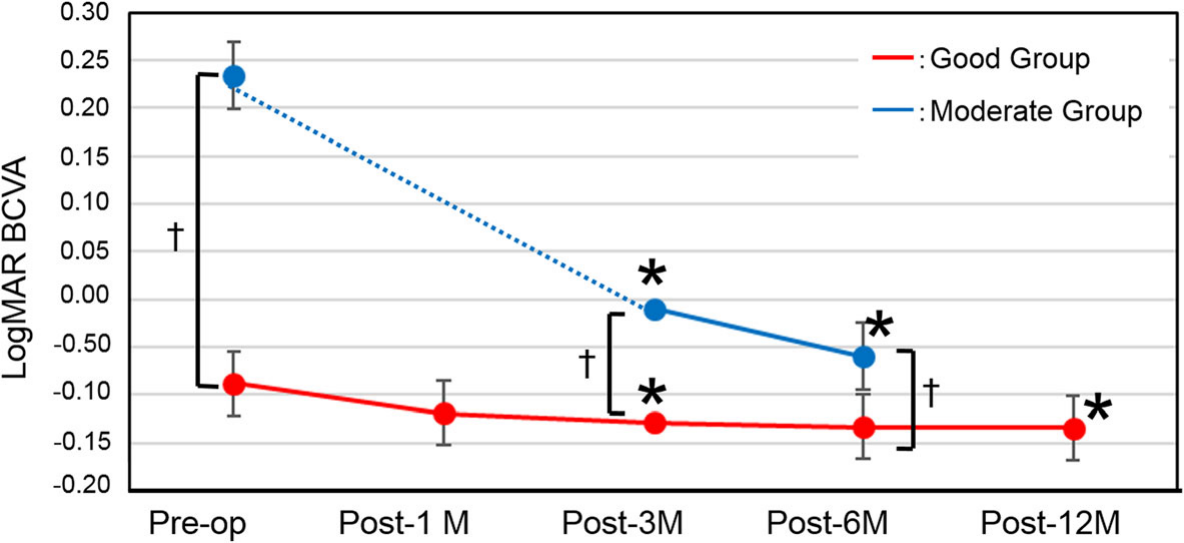
Change in best-corrected visual acuity (BCVA) (logMAR). Red line: Good group, Blue line: Moderate group. *P < 0.05 compared with preoperative; †*P* < 0.05 between groups. Because the data obtained at one month were missing from the Moderate group, the dotted line indicates the period between the preoperative and post-6 M time points

### Horizontal metamorphopsia scores (Table 2, Fig. 2)

The scores at post-1 M, post-3 M, post-6 M, and post-12 M in the Good group indicated significant improvement of MH at each time point (*P* = 0.0003, *P* < 0.0001, *P* < 0.0001, and *P* < 0.0001, respectively). In the Moderate group, the scores at post-3 M and post-6 M showed significant improvement (*P* = 0.015 and *P* < 0.0001, respectively). However, there were no significant differences between two groups at post-3 M and post-6 M.

**Fig. 2:**
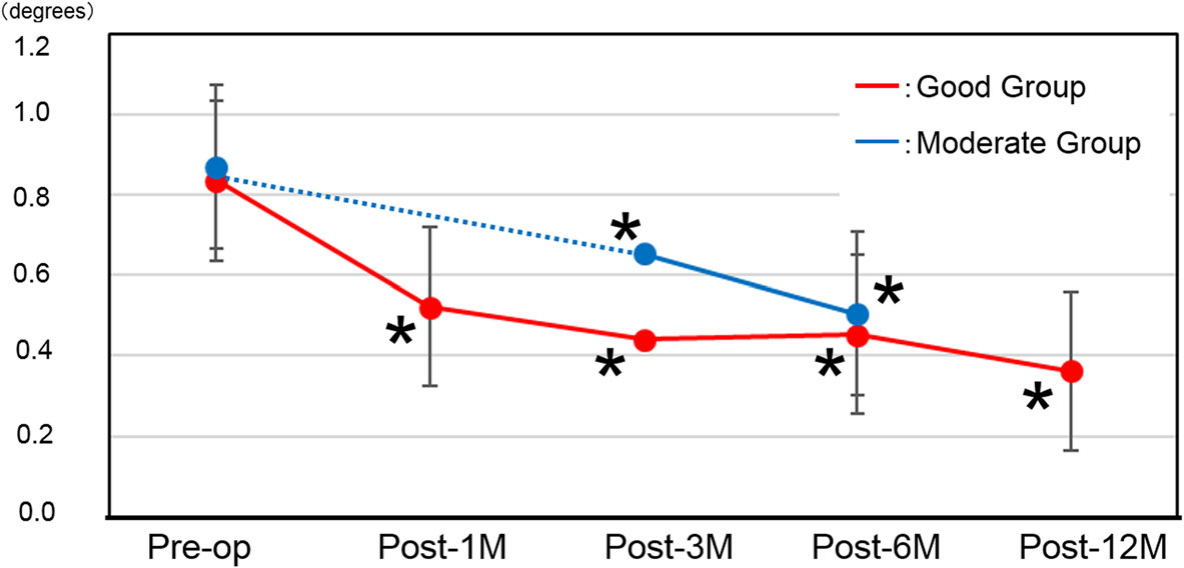
Horizontal metamorphopsia scores. Red line: Good group, Blue line: Moderate group. *P < 0.05 compared with preoperative score

### Vertical metamorphopsia scores (Table 2, Fig. 3)

The scores at post-1 M, post-3 M and post-6 M in the Good group all showed significant improvement in MV (*P* = 0.0006, *P* = 0.048, and *P* = 0.018, respectively). In the Moderate group, only the score at post-3 M showed significant improvement. However, there were no significant improvements at the final observation in either group (*P* = 0.38 at post-12 M in the Good group and *P* = 0.06 at post-6 M in the Moderate group). There were no significant differences between the two groups preoperatively or at post-3 M or post-6 M (*P* = 0.62, *P* = 0.22, and *P* = 0.85, respectively).

**Fig. 3:**
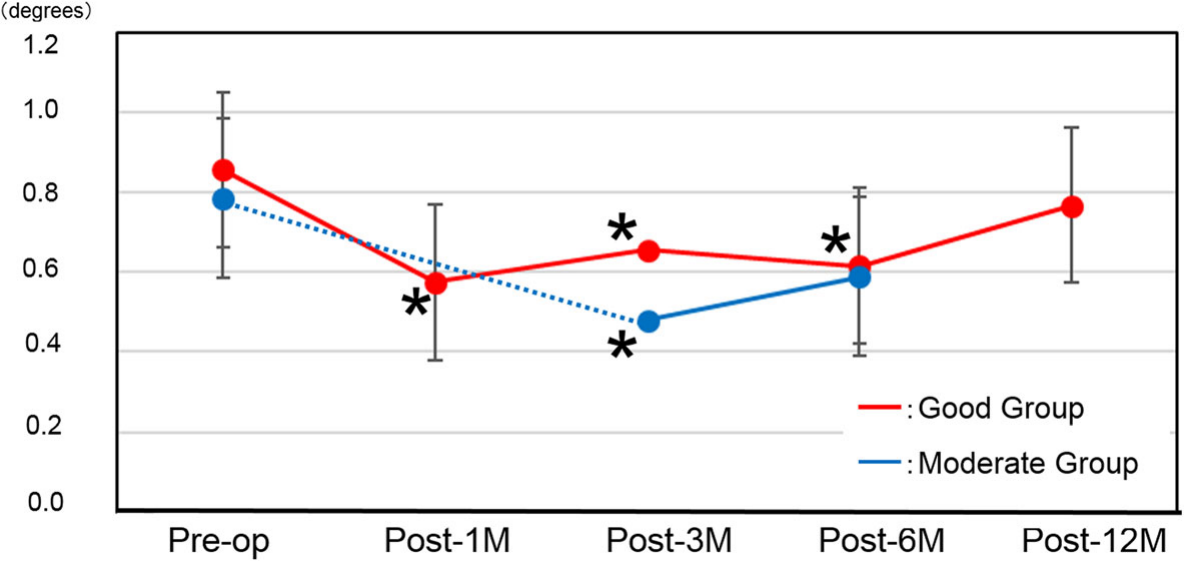
Vertical metamorphopsia scores. Red line: Good group, Blue line: Moderate group. *P < 0.05 compared with preoperative score

### Horizontal aniseikonia scores (Table 2, Fig. 4)

The Good group showed no changes in AH as compared with the preoperative rate at any of the observation points. In the Moderate group, significant worsening of macropsia was seen at post-3 M and post-6 M (*P* = 0.013 and *P* = 0.009, respectively). Though there were no significant differences between the groups at the preoperative time point (*P* = 0.51), in the Good group, significant reductions in macropsia were observed at post-3 M and post-6 M (*P* = 0.005 and *P* = 0.007, respectively).

**Fig. 4:**
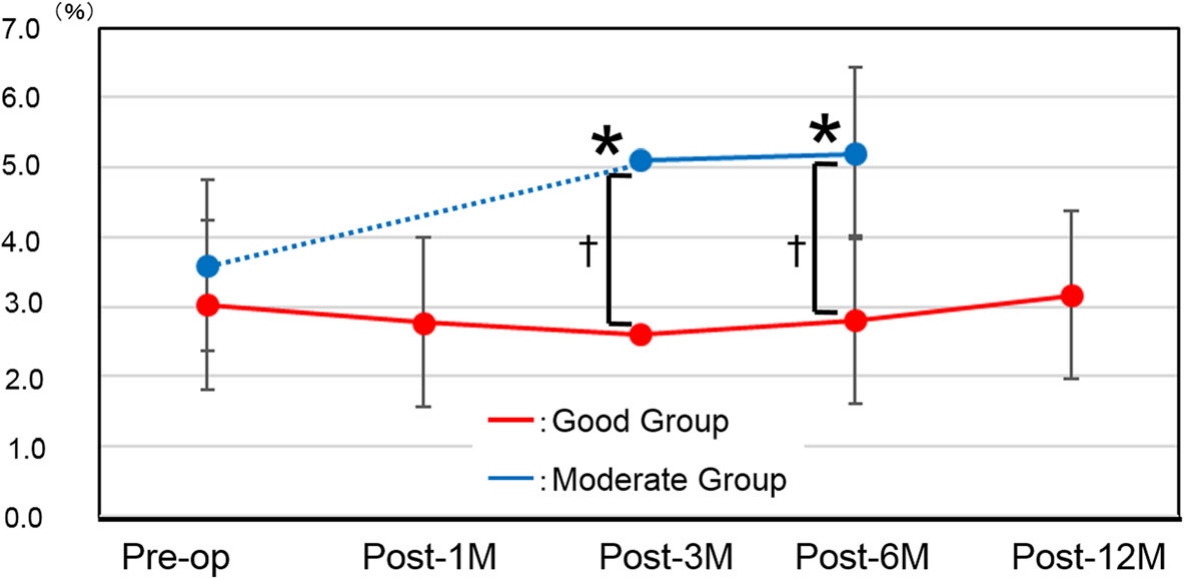
Horizontal aniseikonia scores. Red line: Good group, Blue line: Moderate group. *P < 0.05 compared with preoperative score; †*P* < 0.05 between groups

### Vertical aniseikonia scores (Table 2, Fig. 5)

The Good group showed neither significant improvement nor worsening of AV as compared with the preoperative value at any observation point. In the Moderate group, there was significant worsening of macropsia at post-6 M (*P* = 0.011). Though the two groups did not differ significantly at the preoperative time point (*P* = 0.15), in the Good group, macropsia was significantly reduced at post-3 M and post-6 M (*P* = 0.004 and P = 0.004, respectively).

**Fig. 5:**
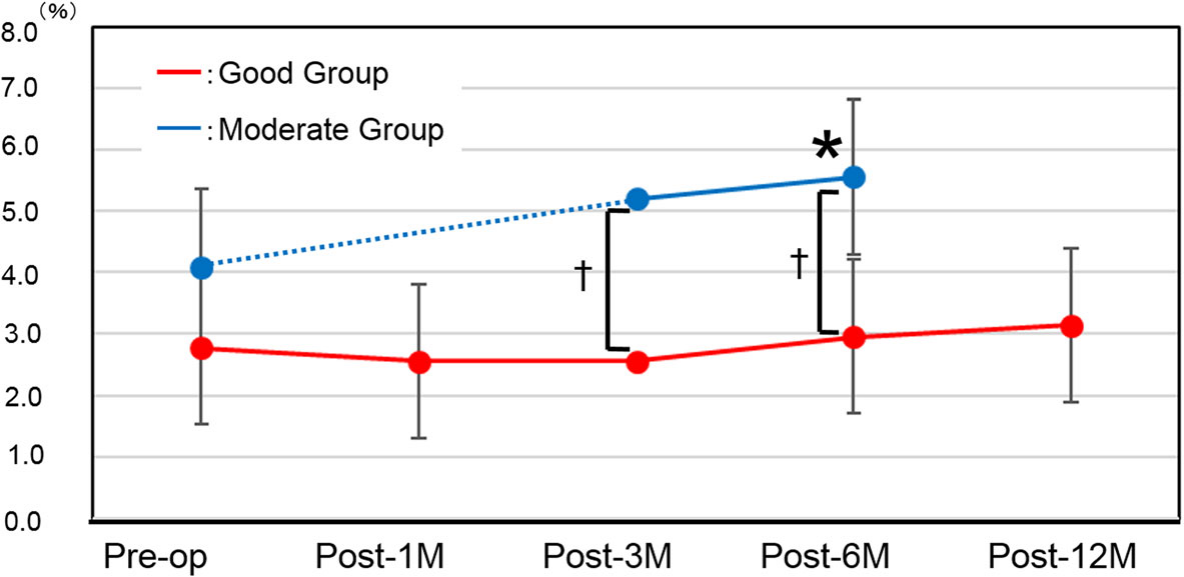
Vertical aniseikonia scores. Red line: Good group, Blue line: Moderate group. *P < 0.05 compared with preoperative score; †*P* < 0.05 between groups

### Binocular vision (Table 2, Fig. 6)

The Good group showed no change in binocular vision as compared with the preoperative value. In the Moderate group, the post-6 M values showed significant improvement (*P* = 0.014). Although intergroup comparison revealed stereopsis to be significantly better in the Good group preoperatively (*P* = 0.02), no significant intergroup differences were found at either post-3 M or post-6 M (*P* = 0.18 and *P* = 0.63, respectively).

**Fig. 6:**
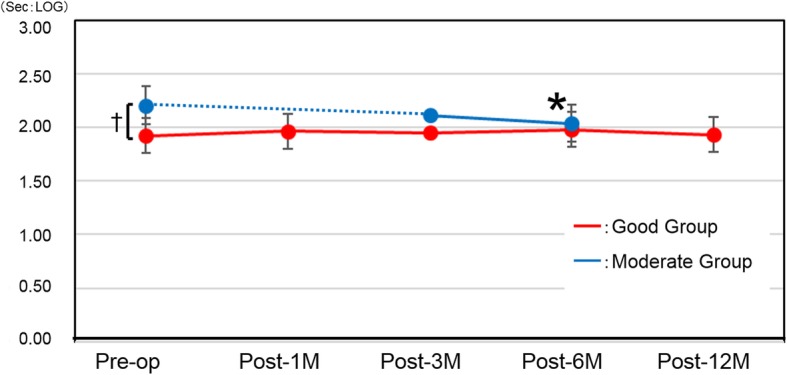
Stereopsis (TST). Red line: Good group, Blue line: Moderate group. *P < 0.05 compared with preoperative score; †*P* < 0.05 between groups

### CFT measurements (Table 2, Fig. 7)

In the Good group, the CFT measurements at post-1 M, post-3 M, post-6 M, and post-12 M indicated significant thinning (*P* = 0.001, *P* < 0.0001, and P < 0.0001). In the Moderate group, the postoperative values at post-3 M and post-6 M showed significant thinning. (*P* < 0.0001 and P < 0.0001). Intergroup comparisons indicated that at the preoperative and post-3 M time points, CFT was significantly thinner in the Good group than in the Moderate group (P < 0.0001 and 0.02), whereas there was no significant difference at the post-6 M time point (*P* = 0.065).

**Fig. 7:**
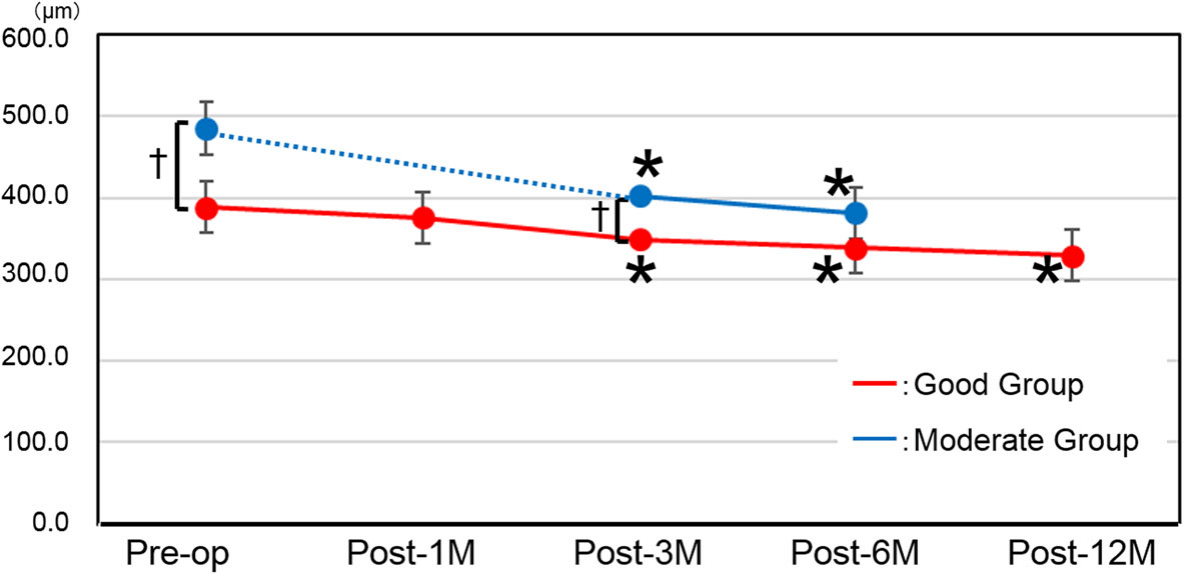
Central foveal thickness. Red line: Good group, Blue line: Moderate group. *P < 0.05 compared with preoperative score; †*P* < 0.05 between groups

### Factors affecting changes in horizontal metamorphopsia score (multivariate analysis) (Table 3)

Factors affecting changes in postoperative MH, including age, preoperative BCVA, preoperative MH, preoperative MV, preoperative AH, preoperative AV, and preoperative CFT, were investigated. Multivariate analysis identified only preoperative MH as a factor that affected changes in postoperative MH. (*P* < 0.0001).

**Table 3:** Factors affecting postoperative horizontal metamorphopsia score (linear mixed-effects model, multivariate analysis)

Trend model	Parameter 95% CI				
	Est.	Lower limit	Upper limit	SD	*P*-value
Segment	−0.0085	−0.1730	0.1560	0.0825	0.9182
Time point (Trend)	0.0334	−0.0150	0.0818	0.0246	0.1757
Pre-op MH	0.9424	0.7878	1.0970	0.0784	< 0.0001
Pre-op MH and Time point (Trend) interaction	−0.1673	−0.2136	−0.1209	0.0235	< 0.0001

*MH* horizontal metamorphopsia score, *SD* standard deviation, *Est*. estimated value

### Horizontal metamorphopsia score variation (Fig. 8)

**Fig. 8:**
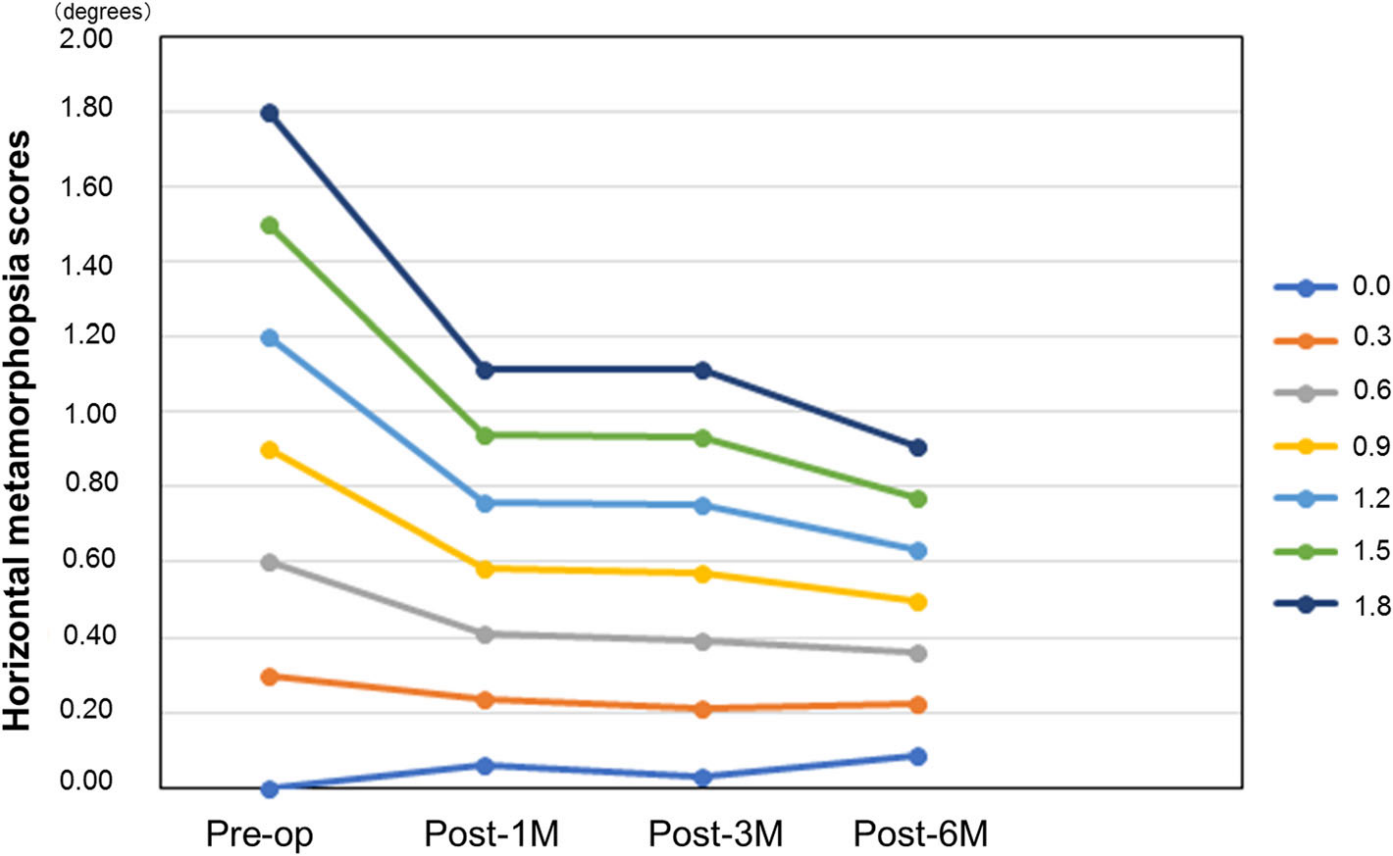
Changes in horizontal metamorphopsia score (using category model). The estimated value of MH at each month of observation was created by the category model (Table 4). The post-6 M metamorphopsia score expected based on preoperative horizontal metamorphopsia score of 0.9° is estimated to be 0.4997° (yellow line)

The estimated MH values obtained by applying the category model (Table 4) are presented in Fig. 8. The post-6 M score was predicted to be 0.4997° based on the preoperative MH of 0.9°.

**Table 4:** Factors affecting postoperative horizontal metamorphopsia score (linear mixed-effects model, multivariate analysis)

Category model		Parameter 95% CI				
		Est.	Lower limit	Upper limit	SD	*P*-value
Segment		0.0000	− 0.1770	0.1770	0.0888	1.0000
Time point	1	0.0594	−0.1734	0.2923	0.1181	0.6153
Time point	2	0.0317	−0.1513	0.2147	0.0928	0.7332
Time point	3	0.0885	−0.0945	0.2715	0.0928	003416
Time point	0	0.0000				
Pre-op MH		1.0000	0.8341	1.1659	0.0842	< 0.0001
Pre-op MH and Time point interaction	1	−0.4152	−0.6406	−0.1897	0.1144	0.0004
Pre-op MH and Time point interaction	2	−0.3974	−0.5709	− 0.2239	0.0880	< 0.0001
Pre-op MH and Time point interaction	3	−0.5431	−0.7167	− 0.3696	0.0880	< 0.0001
Pre-op MH and Time point interaction	0	0.0000				

Time point 0: preoperative, 1: post-1 M, 2: post-3 M, 3: post-6 M MH, horizontal metamorphopsia score; SD, standard deviation; Est., estimated value

### NEI VFQ-25 (good group only), (Table 5)

The VFQ-25 questionnaire results of the Good group, pre- versus post-operative, are summarized in Table 5.

**Table 5:** The National Eye Institute 25-item Visual Function Questionnaire (VFQ-25) composite score and 12 subscales in the Good group

VFQ-25 questionnaire scale	Preoperative	Post-6 M		Post-12 M	
	Average ± SD	Average ± SD	*P*-value	Average ± SD	*P*-value
General health	45.9 ± 16.2	50.0 ± 14.9	0.09	53.6 ± 16.2	0.004*
General vision	59.5 ± 18.6	66.9 ± 17.5	0.005*	69.1 ± 16.3	0.0005*
Ocular pain	74.3 ± 19.3	83.2 ± 18.7	0.002*	81.1 ± 16.7	0.014*
Near activities	64.2 ± 17.2	67.4 ± 16.7	0.167	76.9 ± 15.1	< 0.0001*
Distance activities	75.4 ± 14.2	77.5 ± 11.4	0.45	79.4 ± 11.6	0.138
Social functioning	87.8 ± 16.3	85.4 ± 15.9	0.373	91.8 ± 10.5	0.178
Mental health	70.4 ± 22.3	76.4 ± 19.3	0.047*	79.8 ± 15.7	0.0023*
Role difficulties	80.4 ± 20.3	79.3 ± 18.9	0.815	82.5 ± 16.1	0.45
Dependency	89.0 ± 14.6	91.2 ± 14.6	0.237	93.1 ± 12.0	0.058
Driving	74.3 ± 18.5	74.2 ± 25.6	0.996	73.4 ± 24.5	0.906
Color vision	93.2 ± 15.2	94.3 ± 10.7	0.649	95.7 ± 9.6	0.332
Peripheral vision	70.3 ± 21.1	72.1 ± 19.0	0.684	77.9 ± 18.0	0.067
Composite score	75.3 ± 14.6	77.7 ± 11.8	0.19	82.0 ± 10.8	0.002*

*significant difference from preoperative score (linear mixed-effect model) SD, standard deviation

General health and Near activities showed significant improvement at post-12 M (*P* = 0.004, P < 0.0001). General vision, Ocular pain and Mental health were significantly improved at both post-6 M and post-12 M (*P* = 0.005, *P* = 0.0005: *P* = 0.002, *P* = 0.014: *P* = 0.047 and P = 0.002, respectively). The postoperative composite score showed significant improvement at post-12 M (P = 0.002).

## Discussion

Our present results indicate that early surgery on patients in the Good group with BCVA ≦0.046 logMAR promoted significantly greater BCVA improvement than in the Moderate group. Epiretinal membrane is known to occasionally develop in younger patients [20]. Individuals in certain occupations and those leading certain lifestyles require a high level of visual function. Therefore, we have consider performing surgery on individuals with good BCVA to be an effective approach to maintaining high-quality visual function in such individuals.

Horizontal metamorphopsia is easily perceived by patients [17, 21]. Because it is more common to encounter horizontally oriented text than vertically oriented text while writing and reading, MH is of major importance. Surgery reportedly results in a greater improvement of MH than of MV [11, 13, 15]. Thus, MH at post-6 M was used as the primary outcome measure in the present study.

Although no significant intergroup difference was found in MV at the final observation time point, possibly due to restricted horizontal displacement [13, 21, 22], MH showed improvement in both groups (Moderate group: 0.5°, Good group: 0.37°) at the final observation time point. Once metamorphopsia reaches 0.5° or worse, the patient reportedly becomes aware of the symptoms [21]. A previous study on quality of vision (QOV) reported that metamorphopsia has a more marked influence on QOV than on BCVA [23]. In the present study, in the Good group, MH improved to 0.37°, i.e. < 0.5°, thereby indicating that it was useful for improving QOV.

Only the preoperative MH was selected as a factor that affected the postoperative MH. The calculation of MH using the category model indicated that the preoperative MH leading to postoperative scores of < 0.5° was 0.9°. This result showed that the preoperative MH of 0.9° represents a clinical data point that can be used as an index for determining surgery indications.

The aniseikonia investigation revealed significant worsening of both AV and AH in the Moderate group at post-6 M and that macropsia was > 5%. The reason for this worsening of postoperative macropsia has yet to be identified. A report on aniseikonia by Okamoto et al also showed that, although there was no significant pre- versus postoperative difference, macropsia was increased postoperatively [24]. However, in the Good group in our study, macropsia remained below 5%, though there was no improvement. Aniseikonia ≧ 5% reportedly indicates a loss of binocular vision [25, 26]. Therefore, in patients with a preoperative value of < 5%, this parameter may provide an index applicable to determining surgical indications for patients with good BCVA.

Concerning binocular function, TST is reportedly associated with preoperative CFT [15]. Preoperative CFT was significantly greater in the Moderate group than in the Good group and preoperative stereopsis was significantly poorer before surgery. However, the significant difference in CFT between the two groups had disappeared at post-6 M, resulting in no significant difference in stereopsis. Asaria et al reported that the longer the symptoms persist preoperatively, the worse the pre- and postoperative stereopsis tends to be [27]. In the present study, the time period from initial symptom onset even in the Good group was unknown. If surgery is performed shortly after symptom onset, then better recovery of visual function including binocular function can be achieved.

VFQ-25 assessment was performed only for the Good group. Okamoto et al, who evaluated patients with a mean preoperative logMAR score of 0.495, reported that the composite score improved from 66.2 preoperatively to 77.9 postoperatively and that all items, with the exception of general health and peripheral vision, showed significant postoperative improvement [9]. The preoperative BCVA was good in the present study. Therefore, the preoperative composite score was also high at 75.3. Despite this, the score at post-12 M showed significant improvement to 82.0, indicating that the surgery in the Good group was effective. Postoperative scores for both general health and vision-related mental health improved, indicating that the patient satisfaction level also improved with the surgery. Both general vision and near activities improved, possibly due to the improvement in visual function itself. As vision itself improved, eye strain was alleviated, which in turn may have ameliorated ocular pain.

The present study prospectively analyzed patients in the Good group. In contrast, because previous data were utilized in the Moderate group, which was used for comparison, postoperative 1 M and 12 M data could not be obtained. Symptoms may also improve at 1 or 2 years postoperatively [28]. Therefore, further investigation of this issue over longer periods of time and with higher numbers of patients is required. The present study included 20 cases with pseudo-macular holes. Although there was no significant intergroup difference in terms of the pseudo-macular hold rate, further investigation without pseudo-macular hole cases should be conducted to investigate metamorphopsia and aniseikonia accurately.

No postoperative complications developed in the present study. However, the BCVA of one 79-year-old male patient with pseudo-macular holes decreased from 1.0 preoperatively to 0.7 in decimal BCVA at 12 M postoperatively. The patient had no subjective perception that his BCVA had declined. It is sometimes difficult to improve BCVA in patients with pseudo- and lamellar macular holes with vitreous traction [29]. Therefore, caution is required when determining surgical indications in such cases with good BCVA. Also, sufficient caution is essential when managing epiretinal membrane patients with uveitis [30], concurrent glaucoma [31], and cyst formation [32].

Recently, micro-incision vitrectomy surgery has come into widespread use and its safety has improved dramatically. Therefore, the surgical indications for macular surgeries have been expanding. Number of patients with epiretinal membrane are expected to increase as the mean age of the population rises. The results obtained in this study indicate that early epiretinal membrane surgery can improve QOL in patients with good BCVA.

## Conclusions

Early epiretinal surgery for patients with BCVA ≦ 0.046 logMAR was effective for improvement of horizontal metamorphopsia, visual acuity, and quality of life and prevented worsening of aniseikonia.

## Data Availability

The datasets used and analyzed during the current study are available from the corresponding author on reasonable request.

## Abbreviations

AH: Horizontal aniseikonia score
AV: Vertical aniseikonia score
BCVA: Best-corrected visual acuity
MH: Horizontal metamorphopsia score
MV: Vertical metamorphopsia score
OCT: Optical coherence tomography
